# FAMILY SUPPORT AS A MEDIATOR OF COGNITIVE FUNCTIONING AMONG HISPANIC OLDER ADULTS WITH SENSORY IMPAIRMENTS

**DOI:** 10.1093/geroni/igac059.2471

**Published:** 2022-12-20

**Authors:** Corinna Trujillo Tanner, Jeremy Yorgason, Avalon White, Joshua Ehrlich, Antonia Cash

**Affiliations:** Brigham Young University, Provo, Utah, United States; Brigham Young University, Provo, Utah, United States; Brigham Young University, Provo, Utah, United States; University of Michigan, Ann Arbor, Michigan, United States; Brigham Young University, Provo, Utah, United States

## Abstract

**Background:**

Understanding the intersection of age, ethnicity, and disability will become increasingly important as the U.S. population ages and becomes more diverse. By 2060, Hispanics, the largest ethnic minority, will comprise 28% of the population. Although a heterogeneous group, originating from a variety of countries, Hispanics may share common cultural values. These familistic values may act as a buffer against social isolation and cognitive decline, commonly associated with sensory disabilities including vision, hearing, and dual sensory disabilities.

**Methods:**

Our sample consisted of 557 Hispanic older adults that participated in the National Health and Aging Trends Study. Longitudinal mediation models across a three-year span were estimated using Mplus with vision, hearing, and dual sensory disabilities predicting cognitive decline directly and indirectly through social isolation. Bootstrapping with 5,000 draws adjusted the standard errors of indirect effects. Results and Discussion: Results suggest that vision disability and dual sensory disability were associated with declines in various cognitive functioning scores. Social isolation was linked with declines in some concurrent and some longitudinal cognitive measures. Although dual sensory disability (i.e., both vision and hearing disability) were linked with social isolation, individual vision or individual hearing disability were not associated with social isolation in this sample. Historically, vision and hearing disabilities have been associated with social isolation, yet in Hispanic cultures, social connections between generations may provide a buffer to this common result of sensory disabilities. Findings from this research suggest that Hispanic older adults experiencing dual sensory disabilities may benefit from interventions that foster social support.

